# Pediatric-onset systemic lupus erythematosus with coronary artery dilation: A case report

**DOI:** 10.1097/MD.0000000000018946

**Published:** 2020-01-31

**Authors:** Hui Zhang, Lijuan Zhang, Nan Guo

**Affiliations:** a The Department of Pediatrics, West China Second University Hospital of Sichuan University; b Key Laboratory of Birth Defects and Related Diseases of Women and Children (Sichuan University), Ministry of Education; c The Ultrasonic Department, West China Second University Hospital of Sichuan University, Chengdu 610041, China.

**Keywords:** children, coronary artery dilatation, echocardiogram, systemic lupus erythematosus

## Abstract

**Introduction:**

Though pediatric-onset systemic lupus erythematosus (SLE) is at high risk of coronary artery involvement, coronary artery dilation appears to be a rare characteristic of pediatric-onset SLE. In this article, we described 1 pediatric-onset SLE patient with coronary artery dilation at the diagnosis of SLE, in order to better diagnose and manage this cardiac complication of SLE in children.

**Patient concerns:**

A 13-year-old boy was admitted in hospital for daily fevers with the highest temperature of 39.2°C over 10 days, with rash, non-exudative conjunctivitis, cervical adenopathy, knee, and ankle arthralgi. The result of echocardiogram implicated coronary artery dilation and aortic regurgitation. Further laboratory tests showed: Coomb's test (+), decreased C3 complement. The results of immunologic tests were only to find ANA (+) with titer 1:3200, ds-DNA (+).

**Diagnosis:**

This patient was diagnosed as SLE complicated with coronary artery dilation.

**Interventions:**

The patient was treated with intravenous methylprednisolone pulse therapy. He was discharged home on prednisone maintain treatment.

**Outcomes:**

As soon as treatment, his temperature returned to normal, with recovery of rash, conjunctivitis, knee, and ankle arthralgi. However, the echocardiogram of this patient after 3 months also had dilation of left coronary artery (LCA) and right coronary artery (RCA).

**Conclusions:**

: Cardiac complication can occur during the entire course of SLE, suggesting that routine echocardiogram surveillance may be necessary for all SLE patients to prevent morbidity and mortality from cardiovascular events.

## 1 Introduction

Systemic lupus erythematosus (SLE) is a complex, relapsing-remitting autoimmune disease that can affect many organ systems, characterized by multiple immunological phenotypes and chronic immune activation. This disease occurs before the age of 18 years in 15% to 20% of SLE patients, with most frequent onset between the ages of 12 and 16 years.^[1,2]^ Unlike other rheumatic diseases such as juvenile dermatomyositis or juvenile idiopathic arthritis, which considerably differ from the adult-onset patients, the clinical features and laboratory findings of pediatric-onset SLE are substantially the same as adults.^[3,4]^ However, pediatric-onset SLE is more acute in onset with more aggressive clinical course, higher rates of major organ involvement and worse prognosis of disease.^[1,5,6]^

Patients with SLE are at high risk of affecting coronary artery, ascribable to some traditional risk factors such as age, gender, family history, hypertension, diabetes mellitus, dyslipidemia, and obesity. Pediatric and adult SLE patients share the similar cardiac manifestations, primarily pericarditis that accounts for about 20% to 30% of SLE patients at onset.^[7]^ Other common cardiac complications in pediatric-onset SLE include myocarditis, valvulitis, and conduction abnormalities. Additionally, left ventricular systolic and diastolic dysfunction, mitral and tricuspid valve insufficiencies, as well as electrocardiograph changes without clinical heart disease, also have been documented.^[7,8]^

Though coronary artery involvement can be frequently found in patients with some other rheumatic disease, such as anti-neutrophil cytoplasmic antibodies (ANCA) associated vasculitis, microscopic polyangiitis, and Takayasu arteritis,^[9,10]^ only a few cases have previously reported coronary arteritis in adult-onset SLE.^[11,12]^ While in pediatric-onset SLE, coronary arteritis has been reported as a rare case by autopsy study.^[13]^

In this article, we described 1 pediatric-onset SLE patient with coronary artery dilation at the diagnosis of SLE. This patient had no cardiac history or complaints of heart disease previously. By documenting this case and reviewing the literatures regarding coronary artery involvement in SLE patients, we aimed to better diagnose and manage this cardiac complication of SLE in children.

## 2 Methods

The case was diagnosed with SLE and presented with coronary artery dilatation, selected from the department of Pediatrics in West China Second University Hospital of Sichuan University. The parents of this patient had provided informed consent for publication of the case. This patient underwent echocardiogram including measurements of the internal diameter of the left coronary artery (LCA), and right coronary artery (RCA). Coronary artery dilatation is defined as the internal diameter of coronary artery being larger than normal without a segmental aneurysm.^[8,14]^

## 3 Case report

**Figure 1:**
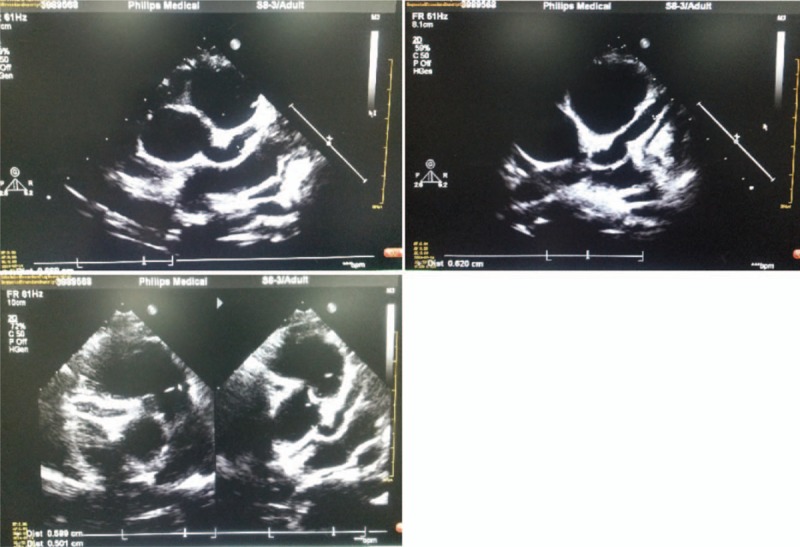
Echocardiogram pictures of case report at the diagnosis of systemic lupus erythematosus (SLE).

A 13-year-old boy was admitted to an outside hospital for daily fevers with the highest temperature of 39.2°C over 10 days. This boy also manifested rash, non-exudative conjunctivitis, cervical adenopathy, knee and ankle arthralgi, without symptoms of chilly, cough, vomiting, abdominal pain, diarrhea, and convulsion. The initial blood routine examination showed: white blood cell (WBC) 3.8 × 10^9^/L, neutrophil% 77.7%, hemoglobin (HGB) 94 g/L, blood platelet (PLT) 322 × 10^9^/L; C-reaction protein (CRP) 82 mg/L; erythrocyte sedimentation rate (ESR) 129 mm/h. Radiographs of the knees, ankles and hands were normal. The result of echocardiogram implicated coronary artery dilation (LCA 5.4 mm, RCA 6.9 mm) and aortic regurgitation (Fig. 1). Because of his persistent fevers longer than 5 days, rash, lymphadenopathy, conjunctivitis, combined with elevated inflammatory markers, echocardiogram findings, he was diagnosed with Kawasaki disease and transferred to the emergency department of our hospital. Physical examination on admission was notable for tachycardia (heart rate 157 beats/min), erythema on his face and hepatosplenomegaly. The results of laboratory tests, including liver function, renal function, coagulation function, and urinalysis, were normal.

In consideration of these clinical findings, differential diagnoses should primarily think about the viral infection, systemic vasculitis, and oncologic diseases. Further laboratory tests showed: Coomb's test (+), blood culture (−), urine culture (−), EBV-IgM (−), decreased C3 complement at 54 mg/dL, normal C4 complement at 22 mg/dL. Bone marrow biopsy was performed and showed normal result. Conventional angiography was negative for vascular abnormalities except for the coronary arteritis. It is noteworthy that the results of immunologic tests were surprising, only to find ANA (+) with titer 1:3200, ds-DNA (+). Thus, this boy was diagnosed as SLE, and treated with intravenous methylprednisolone pulse therapy. As soon as treatment, his temperature returned to normal, with recovery of rash, conjunctivitis, knee and ankle arthralgi. He was discharged home on prednisone maintain treatment. Disappointingly, the echocardiogram of this patient after 3 months also had dilation of LCA and RCA (LCA 5.2 mm, RCA 6.1 mm).

## 4 Discussion

The case with coronary artery dilatation at the onset of SLE in pediatric patient is rare. The patient described before had considerably different clinical features and disease course, who did meet the criteria of Kawasaki disease at onset. For this patient, he was initially misdiagnosed as Kawasaki disease because of the same symptoms as Kawasaki disease. However, he had abnormal immunologic markers consistent with SLE. When it comes to echocardiogram, this patient had dramatic findings of coronary artery dilatation, though there was no cardiac history or complaints of heart disease previously. This case put up a question whether other children with coronary artery dilatation may be misdiagnosed as Kawasaki disease. After all, these 2 diseases share similar features in pathogenesis of vascular inflammation.^[15]^

Factually, cases have been reported of patients meeting the criteria of Kawasaki disease, with coronary artery involvement, only to be diagnosed as SLE later.^[15]^ Additionally, there were some documented cases of adult-onset SLE with coronary artery vasculitis.^[11,12]^ In this population, coronary artery involvement appears to be associated with the activity of disease. What calls for special attention is that cardiac manifestations are usually not a key feature at presentation.^[14,15]^ Moreover, in the majority of patients with coronary arteritis had no cardiovascular history or complaints of heart disease previously,^[14]^ as was seen in our case report. A recent study, which investigated the prevalence of pediatric-onset SLE with coronary artery dilation, indicated that children with SLE had higher possibility of left and right coronary artery dilation, with statistical significance compared to those healthy children.^[14]^ However, this study did not point out whether coronary artery dilatation occurred at onset or during the disease course. In another study for pediatric-onset SLE, 35 of the 75 subjects had cardiovascular involvement at onset, though none with coronary artery dilatation at onset. During the disease course, 7 patients developed coronary artery dilatation later.^[13]^

As far as we know, the risk of cardiovascular disease obviously increased in adult SLE patients, and cardiovascular involvement is emerging as an important cause of morbidity and mortality.^[16,17]^ Currently, about 35% of patients with adult SLE died of either cardiovascular or cerebrovascular disease.^[18]^ Owing to systemic inflammation as an independent risk factor for coronary involvement in rheumatic disease, specifically for SLE, pediatric SLE patients may develop coronary vasculitis at onset of the disease.^[14]^ Other non-classic cardiovascular risk factors found in SLE patients include male gender, antiphospholipid antibodies, increased homocysteine levels, and renal injury.^[19]^

As pediatric SLE patients tend to have high risk of coronary artery involvement, reducing the cardiovascular risk is particularly crucial for these patients. Now that, coronary artery dilatation may occur in pediatric SLE patients during the whole course of the disease,^[13,14]^ however, the performance of a screening echocardiogram to assess coronary artery at onset of the disease has been ignored. On the basis of the case we have described, and the review of literatures, we should pay high attention to the importance of earlier screening of the coronary arteries for newly diagnosed pediatric-onset SLE patients. Therefore, once we have found the evidence of coronary artery involvement, prophylactic aspirin or other medicine may be needed in order to reduce the risk of cardiovascular disease in the future.^[17]^

## 5 Conclusion

In conclusion, coronary artery dilation appears to be not a common characteristic of pediatric-onset SLE. However, children have more aggressive clinical course, and higher rates of major organ involvement, these challenges have compelled pediatricians to perform a screening echocardiogram for initial evaluation of the coronary artery. As the literatures reported, fatal cardiac complication can occur during the entire course of the disease, suggesting that routine echocardiogram surveillance may be necessary for all SLE patients to prevent morbidity and mortality from cardiovascular events.

## Author contributions

**Conceptualization:** Hui Zhang.

**Formal analysis:** Hui Zhang.

**Investigation:** Hui Zhang, Nan Guo.

**Methodology:** Hui Zhang.

**Project administration:** Hui Zhang, Lijuan Zhang.

**Supervision:** Hui Zhang.

**Writing – original draft:** Hui Zhang.

**Writing – review & editing:** Hui Zhang.
